# Contrasting Roles of the Multiple Seas in East Asia on Population Divergence of *Smilax sieboldii* (Smilacaceae)

**DOI:** 10.1002/ece3.71851

**Published:** 2025-07-28

**Authors:** Ya‐Lu Ru, Shan‐Shan Zhu, Xin‐Yi Fan, Wen‐Hao Li, Cheng‐Xin Fu, Yun‐Peng Zhao

**Affiliations:** ^1^ State Key Laboratory for Vegetation Structure, Function and Construction (VegLab), MOE Key Laboratory of Biosystem Homeostasis and Protection, and College of Life Sciences, Zhejiang University Hangzhou China; ^2^ State Key Laboratory for Managing Biotic and Chemical Threats to the Quality and Safety of Agro‐Products Ningbo University Ningbo China

**Keywords:** geographic barriers‐corridor, multi‐sea comparative roles, Quaternary sea‐level fluctuations, temperate forest species

## Abstract

Multiple seas in East Asia have played distinct roles during the Quaternary climatic cycles, which have repeatedly isolated and reconnected temperate forest species, while it remains unclear whether their roles differ. In this study, we used *Smilax sieboldii*, a widely distributed species along the eastern coast of East Asia, to simultaneously evaluate the roles of multiple seas, including the East China Sea, the Yellow‐Bohai Sea, the Korea‐Tsushima Strait, and the Taiwan Strait, as geographic barriers and dispersal corridors during historical sea‐level fluctuations. We employed Bayesian clustering analysis and demographic simulations to elucidate the genetic structure and evolutionary history. The effects of spatial or environmental differences on population structure were examined through isolation by distance (IBD) and isolation by environment (IBE) tests. Further, genetic differentiation and gene flow were used as indicators to assess the roles of different seas as barriers or corridors. A pronounced phylogeographic structure was observed in *S. sieboldii* , with populations divided into three distinct gene pools separated by the East China Sea and the Korea‐Tsushima Strait, accompanied by significant genetic admixture at the lineage boundaries. The lineage divergence occurred during the early Quaternary, while secondary contact began in the most recent interglacial period. During population differentiation, the East China Sea and the Korea‐Tsushima Strait acted as effective geographic barriers, whereas the Taiwan Strait and the Yellow‐Bohai Sea functioned more as dispersal corridors and facilitated greater gene flow. Meanwhile, IBD rather than IBE explained the population structure of *S. sieboldii* . To conclude, the phylogeographic patterns of *S. sieboldii* resulted from population isolation and admixture due to sea‐level fluctuations since the Pleistocene, and the spatial scale of a sea largely determined its ecological role among the multi‐sea systems. These findings improved our understanding of how paleoclimate changes and geological transformations have shaped the speciation and diversification of temperate forest species in East Asia.

## Graphical abstract

This study revealed how regional seascapes differentially drove genetic divergence in a deciduous climbing plant, *Smilax sieboldii*. Results support that both the Korea‐Tsushima Strait and the East China Sea acted as significant geographic barriers to historical gene flow in contrast to both the Yellow‐Bohai Sea and Taiwan Strait.

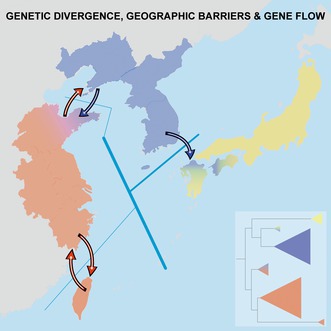

## 1 Introduction

Geographical isolation is a critical mechanism in speciation, beginning with the division of a species into independent populations, followed by the restriction of gene flow and the accumulation of genetic mutations, ultimately leading to the formation of new species over time (Mayr 1942). Geographical barriers (e.g., mountains, rivers, and oceans) are primary drivers of population isolation, while excessive geographical distances and/or environmental differences between allopatric populations will also contribute to the isolation (Baum and Shaw 1995; Coyne and Orr 2004). However, geographical isolation is often disrupted by climate changes and geological transformations, and speciation processes will be accompanied by gene flow and introgression (Coyne and Orr 2004; Feder et al. 2012). Therefore, understanding the respective intensities and dynamic equilibria of isolation and gene flow, and the operation of geographical and environmental factors, is essential for elucidating speciation and biodiversity formation.

**FIGURE 1:**
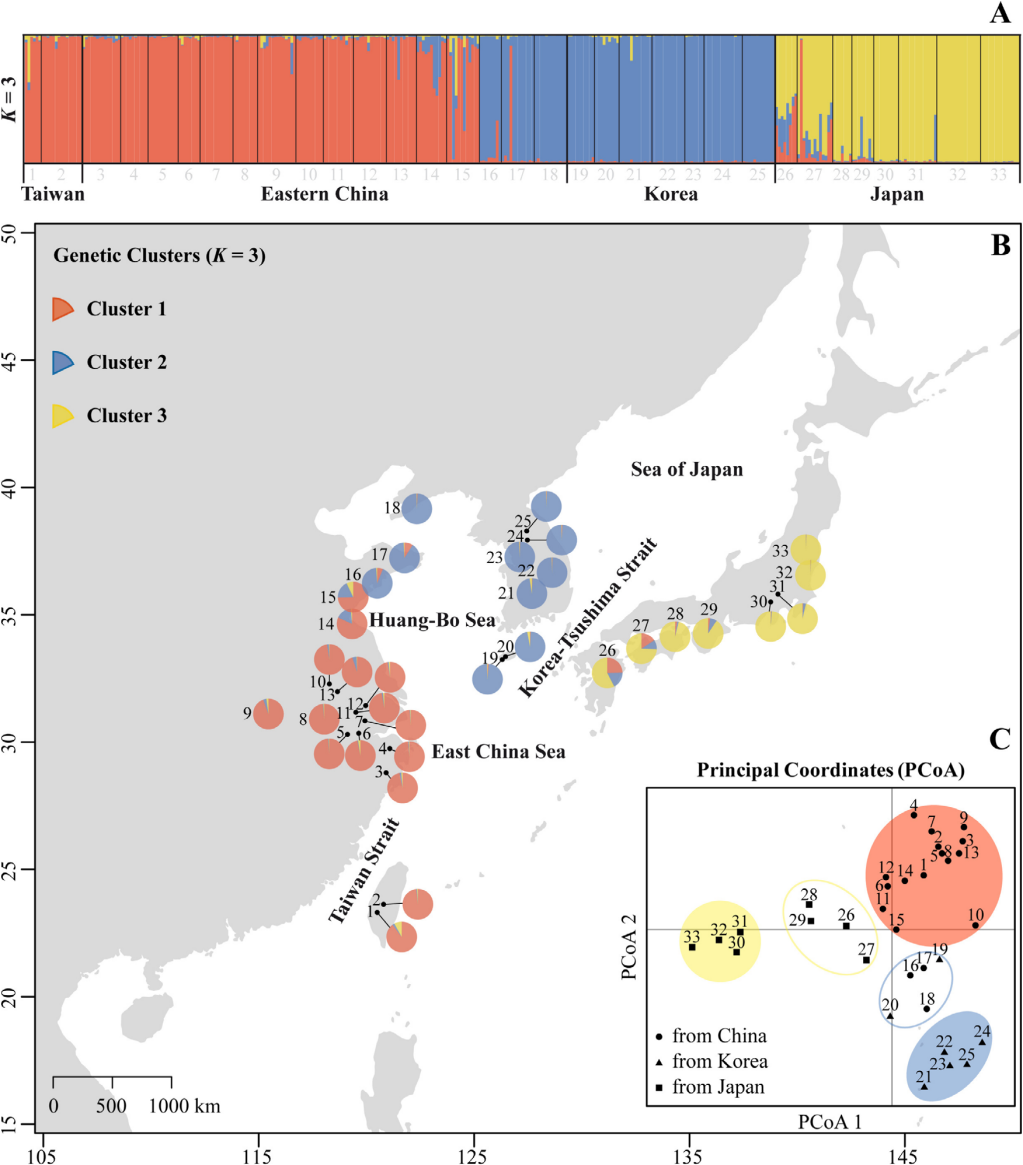
Bar plots (A) and geographical distributions (B) of the genetic assignments of *Smilax sieboldii* at *K* = 3 inferred with the Bayesian method implemented in STRUCTURE. Each individual is represented by a vertical bar and pie chart signifying the proportion of genetic variation belonging to the three different clusters. (C) PCoA of population structure based on SSR data.

Quaternary climate changes and the associated sea‐level fluctuations have led to repeated isolation and contact of regional biota, which has shaped the divergence and genetic patterns of temperate forest species in East Asia (Hewitt 1996; Qian and Ricklefs 2000; Harrison et al. 2001; Qiu et al. 2011; Fu and Wen 2023). The eastern part of East Asia is characterized by the fragmented topography at the continental edge, and it includes the areas of eastern mainland China, the peninsulas of Liaoning (in northeastern China) and the Korean Peninsula, along with the oceanic islands of Taiwan Island, the Ryukyu Islands, and the Japanese Archipelago. These separated areas are now separated by the East China Sea (ECS), the Yellow‐Bohai Sea, the Taiwan Strait, and the Korea‐Tsushima Strait, resulting in discontinuous distributions of the temperate forests (See details in Figure 1). The areas were connected during the Last Glacial Maximum (LGM), when sea levels were at their lowest and the seabed was exposed (otherwise known as land bridges) (Siddall et al. 2003; Spratt and Lisiecki 2016). Despite extensive biogeographic studies in this region, the roles of these seas as either “geographical barriers” or “dispersal corridors” remain debated (Qiu et al. 2011; Jiang et al. 2021; Fu and Wen 2023). For instance, Harrison et al. (2001) collected compelling evidence from pollen and fossil data and proposed that temperate forests in eastern East Asia migrated southward during the LGM, forming a continuous distribution between southeastern China and southern Japan via the exposed ECS land bridge. However, recent genetic data suggested limited gene flow or migration events during the ice age for some key species, indicating that the role of the ECS land bridge as a dispersal corridor during the LGM may have been overestimated (Qiu et al. 2011; Qi et al. 2014). One aspect that is often neglected is that the multiple seas in eastern East Asia are frequently regarded as a unified entity in previous studies, neglecting their distinct characteristics in terms of geographical extent and depth, or even ocean currents, wind, and climatic conditions (Shi 2002; Mei et al. 2016; Hong et al. 2019). The extent of isolation imposed by these seas would determine the potential divergence boundaries and migration routes of the temperate forests, thus a systematic comparative study will help to address this long‐standing controversy and clarify the mechanisms underlying the formation of biodiversity distribution patterns in East Asia.

In this study, we focus on *Smilax sieboldii* (Smilacaceae), a deciduous woody vine distributed widely along the coastal areas of eastern East Asia. It is a dioecious species with unisexual individuals producing clustered umbels of small, yellowish‐green flowers from May to June, exhibiting morphological adaptations for entomophily. The globose berries (6–7 mm diam.), maturing in autumn, display typical ornithochorous syndromes including fleshy pericarp and small seeds (Flora of China; Raven and Axelrod 1974; Zhao et al. 2013). Our field observations documented its strict occurrence in perennially humid, sunlit understories of temperate deciduous forests. Despite an elevational segregation between southern (high‐altitude) and northern (low‐altitude) populations, microclimatic measurements reveal congruent thermal conditions throughout its range. Its populations are now isolated by the multiple seas mentioned above, while lowered sea levels presumably formed terrestrial corridors for population connectivity. Therefore, it provides an ideal model for a comparative test of the “barrier” versus “corridor” effects of the multiple shallow seas. Meanwhile, as a typical understory component of temperate deciduous forests with relatively conservative ecological niches, the population dynamics of *S. sieboldii* should to some extent reflect the migration patterns of temperate forests in East Asia (Zhao et al. 2013; Qi et al. 2023).

Previous phylogeographic studies based on chloroplast DNA revealed at least four geographical lineages of *S. sieboldii* , suggesting divergence due to isolation (Zhao et al. 2013). However, the limited information from a few fragments and the shared haplotypes made it difficult to clarify phylogenetic relationships between the lineages and trace the complex population history of the species (Zhao et al. 2013). Here, we obtained both nuclear simple sequence repeats (SSRs) and single nucleotide polymorphism (SNP) data from populations across the species' distribution range to address the following questions: (1) Do the genetic patterns of *S. sieboldii* align with the distribution of geographic barriers? (2) How did the multiple seas in eastern East Asia serve as geographic barriers or dispersal corridors in the lineage divergence? (3) Which factor has a greater impact on the genetic patterns: the geographic distance or environmental differences? Meanwhile, we will employ demographic modeling to reconstruct the evolutionary history of *S. sieboldii* . Hopefully, this research could provide more desirable cases and evidence for revealing the factors driving the genetic diversification of temperate forest species in East Asia.

## 2 Materials and Methods

### 2.1 Plant Materials

Thirty‐three populations of *Smilax sieboldii* were sampled according to the species' distribution range. At least six individuals from each population were collected, and two leaves from each individual were dried in silica. Sampling information was provided in Table S1 in the Appendix. A total of 363 individuals were used for DNA extraction and microsatellite loci amplification. To provide a phylogenetic framework and information on lineage divergence times for subsequent demographic modeling, we supplemented restriction site‐associated DNA sequencing (RAD‐seq) for a subset of samples. The relevant plant materials, research methods, and results for the RAD‐seq dataset were provided in the Appendix

### 2.2 DNA Extraction, PCR, and Data Processing

Total genomic DNA was extracted from leaves using Plant DNAzol (Invitrogen Life Technologies, California, USA) following the manufacturer's protocol. Polymerase Chain Reaction (PCR) amplifications were conducted on samples of *S. sieboldii* at the 15 polymorphic microsatellite loci developed by Ru et al. (2017). The reaction solution with a final volume of 10 μL contained 2 μL of DNA, 5 μL of 2× PCR Master Mix (Tsingke Biotech Company, Beijing, China), 0.1 μM of forward primer, 0.1 μM of reverse primer, 0.3 μM of fluorescently labeled universal primer, and 2.5 μL of purified water. The PCR thermal profile involved an initial denaturation at 95°C for 5 min; 35 cycles of 94°C for 40 s, 58°C for 30 min, 72°C for 30 s; and a final 10‐min extension step at 72°C. Amplification with the 15 pairs of microsatellite primers yielded clear bands in gel electrophoresis for all 363 samples.

The lengths of PCR products were analyzed on a 3730xl DNA Analyzer (Applied Biosystems, California, USA) with GeneScan 500 LIZ (Applied Biosystems) as an internal reference. Electrophoresis peaks were scored using *GENEMARKER version 2.2.0* (SoftGenetics, State College, Pennsylvania, USA). These SSR loci were tested using *FREENA* (Chapuis and Estoup 2007) and *GENEPOP version 4.0.7* (Rousset 2008) to exclude loci with a high occurrence of null alleles (> 5%), deviation from Hardy–Weinberg equilibrium, or linkage disequilibrium. Further, those loci deviating from the neutrality test will also be excluded. Ultimately, only 12 loci that met all the criteria were used for subsequent analyses.

### 2.3 Unraveling Genetic Diversity and Population Structure

Genetic diversity statistics were computed using *FSTAT 2.9.3* (Goudet 2001). For each population, we calculated the observed number of alleles (*A*_*O*_), the number of private alleles (*A*_*P*_), allele richness (*R*_*S*_), observed heterozygosity (*Ho*), expected heterozygosity (*He*), and Wright's inbreeding coefficient (*F*_*IS*_). Genetic differentiation among populations was quantified using *F*_*ST*_ (Weir and Cockerham 1984).

To clarify the population genetic structure, *STRUCTURE v2.3.4* (Pritchard et al. 2000) was employed to infer distinct gene pools of *S. sieboldii* and to assign individuals into putative genetic clusters. The SSR dataset was processed, and the program was run under the admixture model without prior population information. Ten replicates per *K* cluster (*K* ranging from 1 to 10) were run for 1,000,000 generations, with the first 250,000 generations discarded as burn‐ins. The optimal number of genetic clusters (*K*) was determined using the *ΔK* method based on Evanno statistics (Evanno et al. 2005), implemented in *STRUCTURE HARVESTER* (Earl and von Holdt 2012). Results of different runs were combined and visualized using *CLUMPP* (Jakobsson and Rosenberg 2007). To demonstrate the geographical distribution of the genetic structure, the assignment of genetic clusters of each population was plotted as pie charts on the map using *R v4.3.1* (www.r‐project.org). In addition, principal coordinate analyses (PCoA) were conducted on the microsatellite data using *GENEPOP version 4.0.7* (Rousset 2008).

### 2.4 Testing Isolation by Distance (IBD) and Isolation by Environment (IBE)

To understand the factors influencing the genetic differentiation patterns among populations of *S. sieboldii* , the effects of isolation by distance (IBD) and isolation by environment (IBE) were tested. The genetic distances among populations were calculated as *F*_*ST*_/(1 − *F*_*ST*_). The geographic distances were calculated as the Euclidean distances based on the coordinates of the populations. The environmental distances between populations were calculated as Canberra distances based on the 19 standard bioclimatic variables of each population site from WorldClim version 2.1 (https://www.worldclim.org/). Based on these calculated distances, the partial Mantel test was performed using the “vegan” package (Dixon 2003) in R. The genetic distance was used as the response, the geographic distance was the predictor in the IBD test, and the environmental distance was the predictor in the IBE test. Meanwhile, linear regression analyses were also performed in R to assess the correlations between these predictors and the response variable.

### 2.5 Comparative Analysis of Roles of the East Asia Seas

To evaluate the roles of multiple seas as geographical barriers in population differentiation, we employed two indicators: the coefficient of genetic differentiation (*F*_*ST*_) and the intensity of gene flow between the studied populations. *F*_*ST*_ reflects the degree of genetic differentiation among populations, whereas gene flow determines the strength of genetic connectivity between them. Elevated *F*_*ST*_ coupled with restricted gene flow typically indicates long‐term isolation, which may lead to genetic drift and local adaptation. Therefore, it can be deduced that stronger isolation effects typically result in higher genetic differentiation and reduced gene flow between populations on either side of the geographic barrier, whereas dispersal corridors exert opposing effects. Pairwise gene flow among the populations was calculated using *MIGRATE version 4.0* (Beerli et al. 2019). The values of these indicators were categorized based on the seas separating the tested populations. The categories included “within the land,” “isolated by the Yellow‐Bohai Sea,” “isolated by the Taiwan Strait,” “isolated by the Korea‐Tsushima Strait,” and “isolated by the East China Sea.” For each pair of *F*_*ST*_ and gene flow groups, a Wilcoxon test was conducted to determine whether they were statistically significantly different from one another.

### 2.6 Inferring Population Demographic History

To infer the demographic history of *S. sieboldii* , we employed the Approximate Bayesian Computation (ABC) framework implemented in *DIY‐ABC v.2.1.0* (Cornuet et al. 2014) using the SSR dataset. The results from the preceding analyses had served as the foundation for designing the models. The STRUCTURE analyses indicated an initial divergence between populations in eastern China, Korea, and northern Japan, with admixed populations observed at the intersection of clusters, suggesting secondary contact between divergent lineages (Figure 1; refer to the Results section for detailed information). However, it remains unclear what the specific route for secondary contact is and when these events occurred. To address this, we designed three scenarios to test alternative hypotheses regarding the time and routes of lineage divergence and contact. Scenario 1 represented a basic model of sequential population divergence from east China to north Japan. Scenario 2 assumed secondary contact via a north route across the Yellow‐Bohai Sea and the Korea‐Tsushima Strait. Scenario 3 considered a south route with direct contact between the Chinese and Japanese lineages across the East China Sea, potentially facilitated by a land bridge. Graphical representations of these scenarios are provided in Figure 4.

The 33 populations were divided into five groups based on their genetic clusters and geographic distributions: Group 1 included populations with a red gene pool from southeastern mainland China and Taiwan Island; Group 3 comprised populations with a blue gene pool from Korea and the adjacent Liaoning and Shandong provinces in northeastern China. Group 5 consisted of pure yellow populations from northern Japan. A population was classified as admixed if its major genetic component accounted for less than 75% of its genetic makeup. Thus, admixed populations in northern China were assigned to Group 2, while those with yellow‐red‐blue gene pools in southern Japan were assigned to Group 4 (see Figure 1 for detailed population assignments and Figure S1 in Appendix for phylogenetic relationships among populations).

Prior distributions for divergence times (measured in generations) were set based on the results of BEAST analyses: a uniform distribution of 8.0 × 10^5^–2.0 × 10^6^ for *T*_*d*1_ and 6 × 10^5^–1.7 × 10^6^ for *T*_*d*2_ (see Figure S2 in Appendix for detailed BEAST analyses results). Prior distributions for the formation times of admixed populations were set to uniform distributions of 10–2.0 × 10^6^ for *T*_*m*1_ and *T*_*m*2_. We set *T*_*d*1_ > *T*_*m*1_ > *T*_*d*2_ > *T*_*m*2_ in Scenario 1, and *T*_*d*1_ > *T*_*d*2_ > *T*_*m*1_ and *T*_*m*2_ in the other scenarios. Population sizes (*N*_group1_ − *N*_group5_) were assigned a wide uniform distribution of 10–2.0 × 10^6^, and the admixture rates, *R*_*a*_ and *R*_*b*_, were set to a uniform distribution of 0.001–0.999.

All the 12 SSR loci were pooled into one group, and a generalized stepwise mutation model was applied. The mean mutation rate was initially set to the default range of 10^−4^–10^−3^ mutations per locus per generation. After the first round of simulations, which showed a bias toward lower mutation rates, the range was adjusted to 10^−5^–10^−3^. One million simulations were run for each scenario, and each simulation was summarized using all available summary statistics. Next, the scenarios were compared based on posterior probabilities calculated using a local logistic regression on 1% of the simulated datasets closest to the observed data. Then, the posterior distributions of historical demographic parameters were estimated for the most likely scenario. Finally, the reliability of the selected model was evaluated through model checking and by computing the confidence in scenario choice.

## 3 Results

### 3.1 Nuclear Microsatellite Diversity and Population Structure

The 12 SSR loci exhibited high polymorphism, with the number of alleles ranging from 25 to 70, the allelic richness from 1.82 to 4.27, the observed heterozygosity from 0.30 to 0.81, the expected heterozygosity from 0.24 to 0.69, and Wright's inbreeding coefficient from −0.3718 to −0.002. Private alleles were found in the populations in eastern China, southern Korea, and southern Japan (the specific values for each population are provided in Table S1 in Appendix).

In STRUCTURE analyses, Evanno's statistics revealed an optimal value of *K* = 3 (Figure S3 in Appendix), assigning *S. sieboldii* to three distinct genetic clusters. The geographical distributions of the gene pool assignments showed an intuitive pattern with a “Red” gene pool in eastern China, including Taiwan Island, a “Blue” gene pool in Korea and in northeastern China (Liaoning), and a “yellow” gene pool in northern Japan. While populations from the lineage boundaries in China (Shandong & Jiangsu) and southern Japan showed extensive admixture and were assigned to all three gene pools with varying contributions (Figure 1A,B). Accordingly, the PCoA analyses exhibited a similar pattern consistent with geographical distributions (Figure 1C).

### 3.2 Effect of IBD and IBE

**FIGURE 2:**
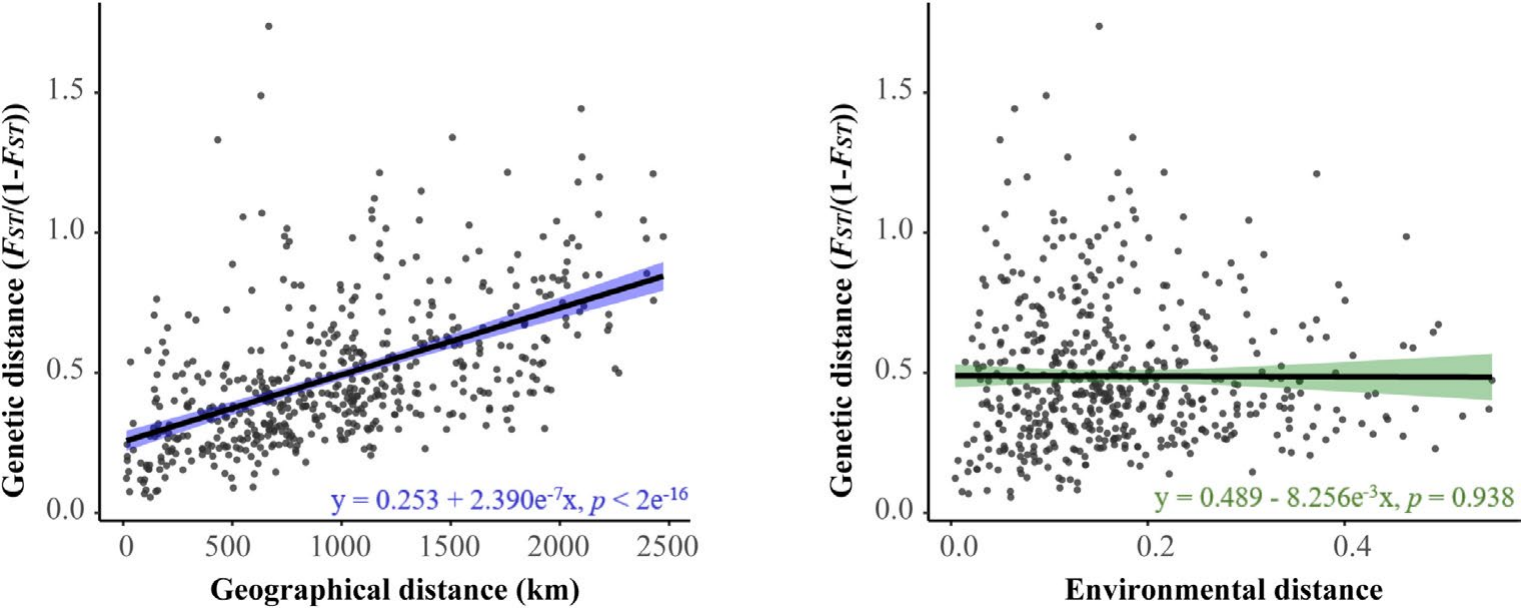
Linear regression analyses of genetic distance in relation to geographic and environmental distances. Results of (A) Isolation by Distance (IBD) and (B) Isolation by Environment (IBE) test. Mantel and Partial Mantel tests revealed a significant correlation between genetic and geographic distances, but not between environmental distances.

Results of the Mantel test suggested that the population genetic structure of *S. sieboldii* could be explained by IBD (*r* = 0.5408, *p* = 1e^−4^) rather than IBE (*r* = −0.003391, *p* = 0.4562) (Table 1). The Partial Mantel test revealed a significant positive correlation between genetic distance and geographic distance (*r* = 0.557, *p* = 1e^−4^) while controlling for environmental distance (Table 1). Meanwhile, the linear regression analyses revealed a significant positive relationship between genetic distance and geographic distance (*β* = 0.2534, *p* < 2e^−16^, *R* ^*2*^ = 0.2925) (Figure 2). The overall model fit was statistically significant (*F*‐statistic = 217.5, *p* < 2e^−16^), with geographic distance explaining approximately 29.25% of the variance in genetic distance. Residual analyses indicated no significant deviations from the assumptions of normality, homoscedasticity, or independence, suggesting that the model is appropriate for the data (Figure S4 in Appendix). The strength of the relationship suggested that geographic distance played an important role in driving population divergence of *S. sieboldii* .

**TABLE 1:** Statistical results of isolation by distance (IBD) and isolation by environment (IBE) tests.

Test type	Variable pair	Correlation (*r*)	*p*	Significance
IBD	Genetic vs. geographic distance	0.5408	0.0001	***
IBE	Genetic vs. environmental distance	−0.0034	0.4562	n.s.
IBD + IBE	Genetic vs. (geographic + environmental) distance	0.2335	0.0042	***

*** Indicates a *p*‐value ≤ 0.001, representing extremely statistically significant.

### 3.3 Contrasting Effects of the Multiple Seas in East Asia: Geographical Barriers vs. Dispersal Corridors

**FIGURE 3:**
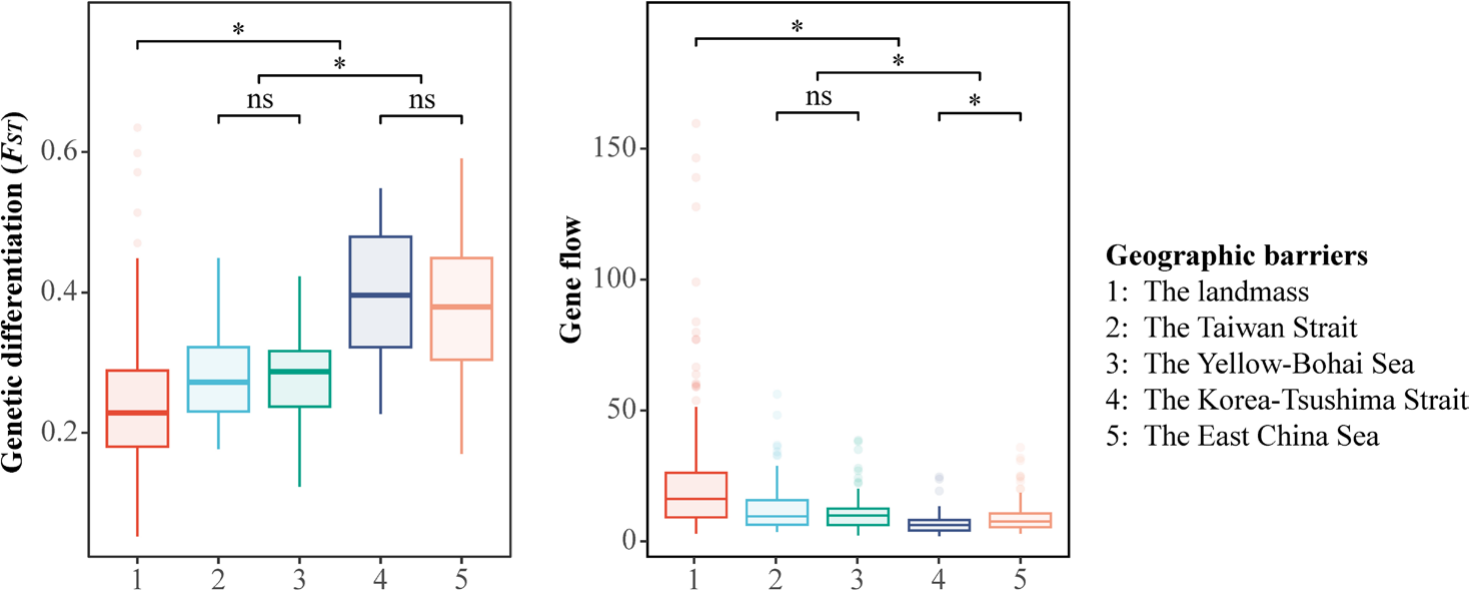
Comparison of genetic differentiation and gene flow across *Smilax sieboldii* groups represented by box plots. Wilcoxon rank‐sum tests were performed for each two groups (*: significant, ns: not significant).

To evaluate the isolating effects of different geographic barriers, we compared the strength of genetic differentiation (*F*_*ST*_) and gene flow between populations separated by various barriers using the Wilcoxon rank‐sum test with continuity correction. The results revealed significant differences in both predictors considering the groups divided by geographic barriers (Figure 3). First, all sea barriers exhibited stronger isolating effects than land barriers in terms of promoting genetic differentiation and limiting gene flow (mean *F*_*ST*_ = 0.340 vs. 0.244, mean gene flow = 9.279 vs. 22.810, all *p* < 0.05) (Figure 3, Table 2). Meanwhile, the Korea‐Tsushima Strait showed the strongest isolating effect (mean *F*_*ST*_ = 0.398, mean gene flow = 6.352), followed by the East China Sea (mean *F*_*ST*_ = 0.379, mean gene flow = 8.314) (Figure 3, Table 2). Both of these were significantly stronger than the Taiwan Strait (mean *F*_*ST*_ = 0.286, mean gene flow = 13.417) and the Yellow‐Bohai Sea (mean *F*_*ST*_ = 0.280, mean gene flow = 9.822) (all *p* < 0.05) (Figure 3, Table 2). These findings suggest that the multiple sea barriers in East Asia play distinct roles in shaping population genetic structure, with the Korea‐Tsushima Strait and the East China Sea acting as particularly effective geographic barriers.

**TABLE 2:** Genetic differentiation (*F*_*ST*_) and gene flow between populations isolated by multiple geographic barriers.

Geographic barriers	Mean *F*_*ST*_ ^a^	Mean gene flow
The landmass	0.244	22.810
The Taiwan Strait	0.286	13.417
The Yellow‐Bohai Sea	0.280	9.822
The Korea‐Tsushima Strait	0.398	6.352
The East China Sea	0.379	8.314

^a^ *F*_*ST*_: genetic differentiation.

### 3.4 The Demographic History

**FIGURE 4:**
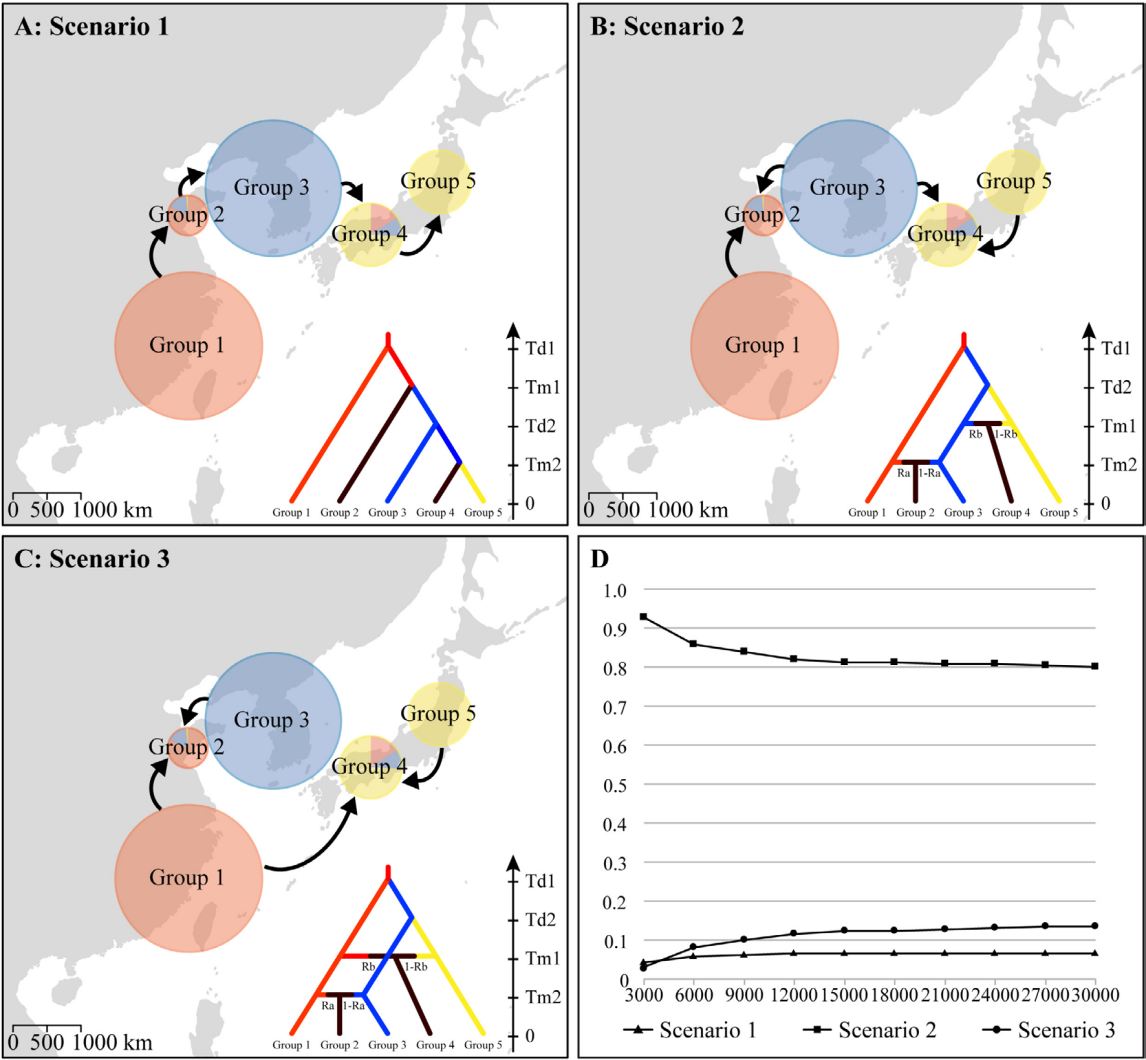
Scenarios of demographic history of *Smilax sieboldii*. (A) A basic model of sequential population divergence from east China to north Japan; (B) Secondary contact via a northern route across the Yellow‐Bohai Sea and the Korea‐Tsushima Strait; (C) Direct contact between the Chinese and Japanese lineages across the East China Sea; (D) Posterior probabilities of each scenario under the logistic approaches. Arrows indicate the directions of migration or gene flow.

The DIY‐ABC analyses provided significant insights into the demographic history and evolutionary processes for the studied populations. Scenario 2, with the highest posterior probability (*PP* = 0.83) outperformed alternative scenarios, as indicated by the logistic regression approach (Figure 4). The confidence in the model choice was further supported by a low posterior predictive error of 0.195 computed over 1000 datasets, which confirmed the robustness of the selected model.

Under Scenario 2, the estimated times of lineage divergence along the East China Sea and the Korea‐Tsushima Strait were approximately 1.48 million years ago (95% credible intervals: 0.81–1.95 Ma) and 1.23 million years ago (95% CI: 0.64–1.77 Ma), respectively. These divergences led to the formation of three distinct genetic lineages in eastern China, Korea, and Japan. Subsequently, the contact between the Korean (45.1%) and Japanese (54.9%) lineages likely occurred 8300 years ago (95% CI: 3500–14,300 years ago), resulting in admixed populations in southern Japan. The admixed populations in northern China originated approximately 7280 years ago (95% CI: 2380–13,500 years ago) due to contact between the eastern Chinese (53.5%) and Korean (46.5%) lineages. The estimates of time assumed a generation time of 2 years for *S. sieboldii* , based on our cultivation experience. The estimated population sizes of the derived lineages were 8940 (95% CI: 7400–9880), 4030 (95% CI: 1200–8290), 5120 (95% CI: 2620–8100), 4310 (95% CI: 1680–8080), and 2020 (95% CI: 836–3870) for group 1 to group 5, respectively. Model checking confirmed a good fit between the observed and simulated data.

The results suggested that *S. sieboldii* experienced a complex demographic history characterized by early lineage divergence followed by very recent secondary contacts. This pattern was consistent with historical climatic fluctuations and habitat fragmentation during the Pleistocene (Hijmans et al. 2005; Qiu et al. 2011).

## 4 Discussion

The region of eastern East Asia provides an ideal system for studying the effects of isolation and migration on plant population dynamics, as its geographic connections, currently separated by seas, have experienced fluctuations associated with Quaternary sea‐level changes and climatic oscillations. Consequently, plant populations in this region have cyclically migrated across exposed seabeds or undergone periods of isolation (Hewitt 1996; Qian and Ricklefs 2000; Harrison et al. 2001). Here, we used *Smilax sieboldii* to compare the influence of the multiple seas on the genetic patterns and population dynamics of temperate forest species in eastern East Asia.

### 4.1 Glacial Refugia and Postglacial Expansion via Northern Coastal Routes Shaped the Phylogeography of *Smilax sieboldii* in East Asia

Microsatellites (SSRs) in *S. sieboldii* appeared to retain the genetic imprint of ancient events. Results from STRUCTURE and ABC simulations suggested that the most likely demographic scenario for the species involved two nearly concurrent divergence events during the Pleistocene (1.23 and 1.48 Ma, respectively), separating the samples into three distinct gene pools (Eastern‐red, Northern‐blue, and Western‐yellow; Figures 1 and 4, Scenario B). This ancient divergence was further supported by the high level of genetic differentiation and the presence of unique private alleles in each lineage (Table S1 in Appendix). Additionally, analyses using chloroplast DNA markers revealed a significant phylogeographic structure in *S. sieboldii* across eastern China, Taiwan, Japan, and Korea (*G*_*ST*_ = 0.820, *N*_*ST*_ = 0.908, *p* < 0.01; Zhao et al. 2013), indicating allopatric speciation and the strong influence of geographic barriers on population divergence. Although most areas of eastern East Asia remained free from extensive ice coverage during the Pleistocene (Shi et al. 1986), vegetation experienced significant retreats and isolations in response to cold climatic conditions (Harrison et al. 2001; Qiu et al. 2011). Initially, temperate forests in East Asia were thought to retreat southward overall during glacial periods, forming refugia between 25°N and 30°N in southern China and southern Japan (Qian and Ricklefs 2000; Harrison et al. 2001). However, this southern refugia model does not apply well to *S. sieboldii* , as its northern lineage from Korea and nearby Liaoning (northern China) exhibited a long evolutionary history and retained high genetic diversity, suggesting a potential refugium in Korea. In addition to the generally accepted southern refugia, a series of cryptic refugia have been identified in northern China, the Korean Peninsula, and northern Japan (Yi and Kim 2010; Qiu et al. 2011; Bai et al. 2016; Aoki et al. 2019; Fu and Wen 2023), supporting in situ survival for many species during glacial periods. Therefore, postglacial population expansions may have followed a radial pattern originating from multiple refugia, rather than a stepwise northward dispersal pattern from the south.

Very recently, secondary contact between diverged lineages occurred 7000–8000 years ago, leading to the formation of admixed populations near the borders of both lineages in northern China and southern Japan (Figure 1). From a temporal perspective, this period coincided with the postglacial era when the conditions became warmer and more humid. It facilitated the rapid expansion of temperate forests, but the sea level began to rise at this point in time. Therefore, the formation of admixed populations in northern China and southern Japan would have resulted from long‐distance overseas dispersal. Given that its seeds are dispersed by birds, such a scenario is plausible for *S. sieboldii* . Importantly, however, these time estimates should be interpreted with caution due to the limitations of microsatellite data, including the uncertain mutation model, homoplasy, and the tendency to underestimate divergence times over large timescales (Takezaki and Nei 1996; Selkoe and Toonen 2006).

It should be noted that the chloroplast‐inferred divergence time was earlier than that estimated from nuclear genes in this study. This discrepancy may arise because (1) the haploid, non‐recombining chloroplast genome has a much smaller effective population size (Ne), leading to faster lineage sorting and thus older divergence time estimates (as reflected in wider 95% credible intervals); and (2) postglacial population expansion might have facilitated nuclear introgression between lineages, compressing the apparent divergence time of nuclear genes. Additionally, while DIY‐ABC estimates divergence times in generations (dependent on generation time assumptions), BEAST relies on molecular clock calibration, which may contribute to numerical differences (see Methods). Nevertheless, both approaches consistently support an Early Quaternary lineage divergence in *S. sieboldii* , followed by secondary contact during the more recent past. In general, the story of *S. sieboldii* should be that relict populations persisted in three or more refugia within the modern ranges of their current lineages during Pleistocene glaciation cycles, and recent recolonization during the postglacial warm climate led to secondary contact of populations along the northern route across the Yellow‐Bohai Sea and Korea‐Tsushima Strait. Similar patterns have been reported for other plants, such as *Kalopanax septemlobus* (Sakaguchi et al. 2012) and Asian butternuts (Bai et al. 2016), and animals like *Hyla japonica* (Dufresnes et al. 2016), and so on. These findings suggest that glacial cycles, although likely milder in East Asia compared to other regions, significantly influenced the demographic processes of species in this area.

### 4.2 The East China Sea and Korea–Tsushima Strait Were Stronger Biogeographic Barriers Than the Taiwan Strait and Yellow–Bohai Sea

During the migration dynamics of plants, geographical barriers shaped the boundaries of lineage divergence, while dispersal corridors determined the routes of recolonization. To evaluate the roles of multiple seas in East Asia as either geographical barriers or dispersal corridors, we compared two datasets: genetic differentiation coefficients and gene flow estimates. Our results indicated that the East China Sea and the Korea‐Tsushima Strait functioned as more effective barriers to population divergence (mean *F*_*ST*_ = 0.379 and 0.398, respectively, compared to 0.286 and 0.280), whereas the Taiwan Strait and the Yellow‐Bohai Sea likely facilitated greater gene flow (mean gene flow = 13.417 and 9.822, respectively, compared to 8.318 and 6.352). Below, we will discuss the specific roles of each sea region in detail.

#### 4.2.1 The East China Sea

The role of the ECS land bridge has been the most extensively discussed and debated. As mentioned in the previous section, early palaeobiome reconstructions suggested that the exposed ECS land bridge during glacial periods served as a dispersal corridor, facilitating the exchange of forest species between eastern China and southern Japan. However, it is important to note that even if the declined sea levels led to the emergence of land bridges, the cold and arid conditions would have been unsuitable for most temperate plant species (Qiu et al. 2011). The ECS land bridge acted as a filter rather than a corridor, with only a few cold‐ and drought‐tolerant species (e.g., *Machilus thunbergii*; Jiang et al. 2021) exhibiting evidence of land bridge connectivity and extensive admixture between eastern China and Japan (Qi et al. 2014). The life history traits and ecological requirements of *S. sieboldii* may not have been conducive to dispersal via the ECS land bridge. Consequently, we observed a distant phylogenetic relationship between the Chinese and Japanese lineages, with no evidence of direct gene flow across the ECS during the LGM.

#### 4.2.2 The Korea‐Tsushima Strait

Our findings indicated that the Korea–Tsushima Strait acted as the most significant geographic barrier among these sea regions, resulting in the strongest genetic differentiation and minimal gene flow. Results of the demographic modeling implied that *S. sieboldii* did not utilize land bridges across the Korea–Tsushima Strait, which connected southern Japan with Korea during the last glacial cycles, and thus remained isolated throughout the Quaternary. The strait, located between Korea and Japan near 35° N, has a width of approximately 180 km and an average depth of about 100 m (Moon and Lee 2016; Shin et al. 2022). The relatively narrow width suggested that additional factors, such as ocean currents or wind dynamics, may have played a significant role in hindering genetic exchange between populations on opposite sides. The impact of ocean currents on genetic patterns of algae and fish in this region has been well documented (Chung et al. 2013; Hu et al. 2015; Zhao et al. 2022). In contrast, wind dynamics likely play a more significant role in shaping the genetic patterns of birds and plants, particularly for bird‐dispersed species like *S. sieboldii* (Bai et al. 2016, 2018). Clustering patterns observed in STRUCTURE analysis indicate population subdivision. Long‐term isolation leads to distinct genetic clusters, whereas hybridization, introgression, and gene flow result in admixed ancestry, where individuals will be fractionally assigned to two or more genetic pools (Pritchard et al. 2000). The admixed populations currently present in southern Japan likely originated from recent secondary contact between the diverged lineages, potentially facilitated by long‐distance dispersal across the sea. Gene flow may have occurred unidirectionally, resulting in no significant genetic consequences for Korean populations while significantly altering the genetic composition of Japanese populations. Within this region, studies on *Rhododendron mucronulatum* (Korean azalea) have documented that Fukue, located midway between Jeju and Kyushu, served as stepping stones for oversea dispersal (Yoichi et al. 2016). But the story varies among species. For example, in the case of tree frogs (Dufresnes et al. 2016), admixed populations formed on the former Sea of Japan land bridge, which subsequently disappeared when water levels rose during interglacial periods.

#### 4.2.3 The Yellow‐Bohai Sea

The north–south divergence of temperate forests in East Asia was primarily driven by an arid belt spanning 35° N to 45° N, which separated the northern region (northeastern China, Korea, and Japan) from the southern region (eastern, southeastern, and southwestern China) (Tiffney and Manchester 2001; Guo et al. 2008). Despite the absence of samples from North Korea, populations of *S. sieboldii* exhibited a clear genetic transition from eastern China to the Korean Peninsula. In Shandong's genetically admixed regions, southern populations had a higher proportion of Chinese genetic components, while northern populations exhibited more Korean genetic components. This pattern appeared to reflect an ongoing admixture process at the expansion front. Demographic modeling indicates that this admixture occurred approximately 8000 years ago, coinciding with the transition from the last glacial period to the interglacial period. Thus, we proposed two scenarios to explain this pattern: first, the Yellow‐Bohai Sea dried up during the glacial period (Kim et al. 1998), forming a continuous landmass that facilitated contact between the southern Chinese lineage and those from the Korean Peninsula; or second, during the interglacial warming, population expansion driven by long‐distance dispersers led to genetic exchange among populations at the expansion front.

#### 4.2.4 The Taiwan Strait

In our study, Taiwan and mainland China shared nearly identical genetic components (Red gene pool, Figure 1), with minimal genetic differentiation and strong gene flow across the Taiwan Strait. However, previous studies based on chloroplast DNA fragments identified unique haplotypes in Taiwan, suggesting an earlier divergence from the mainland groups. SSR markers, as biparentally inherited nuclear markers dispersed by both pollen and seeds, evolve more rapidly than chloroplast markers and thus reflect more recent historical events (Ellegren 2004). We therefore hypothesized that the observed genetic changes in the nuclear genome of populations in Taiwan had resulted from recent high‐intensity gene introgression, particularly through extensive pollen flow between populations, which can erase genetic signatures of Pleistocene refugial isolation (Liepelt et al. 2002). The close relationship between the flora and fauna of Taiwan and mainland China has long been documented (Ying and Xu 2002), and recent molecular evidence further supports ongoing biotic exchanges between the two regions (Chen et al. 2015; He et al. 2018; Cheng et al. 2024). It has been explained by three key factors. First, the Taiwan island emerged above sea level only at the Mio‐Pliocene boundary (5 Ma), and its biota is a young community formed through immigration and in situ speciation. Second, the short distance (130 km) between Taiwan and mainland China has facilitated long‐distance dispersal across the strait. Third, during glacial–interglacial cycles, periodic land bridges between Taiwan and mainland China acted as major dispersal corridors, promoting intermittent biotic exchanges (Sibuet and Hsu 2004; Chen et al. 2015; He et al. 2018; Jiang et al. 2019; Cheng et al. 2024). Another noteworthy point was that *S. sieboldii* exhibited a “sky island” distribution pattern, occurring in high‐altitude mountainous regions in both Taiwan (above 2500 m) and southeastern China (above 900 m). This disjunct distribution has been explained by the “postglacial contraction hypothesis,” which highlights the similarities in mountainous topography and vertical vegetation between these regions (Ian Milne 2006; Chou et al. 2011; Jin et al. 2020). According to this hypothesis, cold‐adapted plants such as *S. sieboldii* were widely distributed at lower elevations across both mainland China and Taiwan during glacial periods. As the climate warmed during the postglacial period, these plants migrated to higher elevations, resulting in their absence in the lower‐altitude areas between these regions (Freeman et al. 2018).

### 4.3 Geographic Distance Mediates the Effects of Seas on Population Divergence in East Asia

One explanation for the differing roles of these multiple seas lies in the varying geographical distances of these barriers. Plants dispersed by birds, such as *S. sieboldii* , were more likely to migrate across the Yellow‐Bohai Sea (The nearest distance is approximately 105 km) and the Taiwan Strait (130 km), where the geographical distances are relatively short. In contrast, dispersal across the East China Sea (600 km), with its greater span, was more challenging. Previous studies on other species (e.g., butterflies: Hirao et al. 2015; ants: Wepfer et al. 2016; soybean: Leamy et al. 2016; *Aristolochia delavayi*: Yu et al. 2021; seagrass: Hosokawa et al. 2025) have also found a significant effect of distance on compositional dissimilarity, indicating that greater distances between two areas reduce the likelihood of gene pool exchanges, regardless of environmental suitability. The limited explanatory power of IBD (*R* ^2^ = 0.2925) indicates influences beyond geographic distance. Our IBE analysis excluded natural selection as a significant driver (*p* > 0.05). Then, one possible explanation is that historical demographic events, including colonization expansion, population contraction, and historical isolation followed by secondary contact, overshadowed the effect of geographic distance. In *S. sieboldii* , isolation during the glacial periods likely drove genetic divergence among now‐proximate populations, while recent avian‐assisted dispersal enhanced long‐distance gene flow, weakening isolation by distance patterns. Additionally, IBD's assumption of linear genetic‐distance relationships may oversimplify reality, as other geographic barriers (e.g., mountains) unmodeled here can strongly disrupt gene flow and reduce IBD's explanatory power. Further discussion is needed to clarify how the enhanced role of seas correlates with linear distances between populations. Although the isolation by distance (IBD) effect explained differentiation among all populations separated by seas and landmasses, genetic differentiation between populations separated by seas was significantly higher than that between populations within the same landmass (Figure 3). Future research should focus on integrating additional environmental variables and conducting more detailed studies on the dispersal mechanisms of *S. sieboldii* and other temperate trees to gain a comprehensive understanding of the evolutionary dynamics in East Asia.

## 5 Conclusions

Genetic and phylogeographic patterns for *S. sieboldii* in East Asia indicated that its geographic distribution and population dynamics resulted from the interplay of Pleistocene lineage divergence caused by glacial isolation and recent secondary contact due to population expansion. More importantly, the comparison of the multiple seas that contributed to population isolation and contact inspired us to explore the key factors driving biogeographic diversity and evolutionary patterns in *S. sieboldii* . These results provided insights into how paleoclimate changes and geological transformations have contributed to the speciation and diversification of temperate forests in East Asia.

## Author Contributions

**Ya‐Lu Ru:** conceptualization (equal), data curation (lead), formal analysis (lead), funding acquisition (supporting), investigation (lead), methodology (lead), validation (equal), visualization (equal), writing – original draft (equal), writing – review and editing (equal). **Shan‐Shan Zhu:** writing – original draft (supporting), writing – review and editing (supporting). **Xin‐Yi Fan:** visualization (supporting), writing – original draft (supporting). **Wen‐Hao Li:** writing – original draft (supporting). **Cheng‐Xin Fu:** writing – review and editing (supporting). **Yun‐Peng Zhao:** conceptualization (lead), funding acquisition (lead), project administration (lead), supervision (lead), writing – original draft (equal), writing – review and editing (equal).

## Conflicts of Interest

The authors declare no conflicts of interest.

## Supporting information


**Data S1:** ece371851‐sup‐0001‐Supinfo.docx.

## Data Availability

The data supporting the findings of this study are available in the Supporting Information of this article. Additional datasets, including RAD‐seq reads, have been deposited in the China National Center for Bioinformation (CNCB) under the project of PRJCA032609 and with GSA number CRA020615. All other relevant data are available from the corresponding author upon reasonable request.
