# Supplementary material for: Contrasting Roles of the Multiple Seas in East Asia on Population Divergence of Smilax sieboldii (Smilacaceae)
Source: Ecol Evol. 2025 Jul 28;15(8):e71851. doi: 10.1002/ece3.71851 (PMC12304436; doi:10.1002/ece3.71851)
Supplement: Supplementary file 1 — Data S1: ece371851‐sup‐0001‐Supinfo.docx. [file ECE3-15-e71851-s001.docx]

**Appendix**

To establish a phylogenetic framework and estimate lineage divergence times for subsequent demographic modeling, we have supplemented the data and analyses here.

**Plant Materials:** For *Smilax sieboldii*, we analyzed one randomly selected individual per population across all 33 populations in our existing SSR dataset (see Methods). Phylogenetic analyses employed *S. scobinicaulis* (4 randomly selected accessions from our voucher specimens) as the outgroup, following the robust phylogenetic evidence provided by Zhao et al. (2013) and Qi et al. (2023).. A total of 37 samples were included, and information of the accessions used herein was available in **Table S1** in Appendix.

**RAD - seq and SNP calling:** RAD libraries were constructed and subjected to high - throughput sequencing at the Beijing Genomics Institute (BGI, Shenzhen, China) following their standardized protocols. Sequencing was performed on the HiSeq 2000 platform. The generated reads were processed and genotyped using the denovo_map.pl pipeline in the STACKS software package (Catchen et al., 2013), with parameters set to M = 4, n = 4, and m = 3. Variant sites were filtered using the "populations" function, retaining SNPs with a minor allele frequency (MAF) > 0.05, a maximum observed heterozygosity of 0.7, and occurring in > 75% of individuals for downstream analyses. A total of 25.3 Gb of raw data was obtained from 35 samples, excluding two samples (JJU and JOM) due to unsuccessful library construction. After quality control, an average of 5,292,018 clean reads per sample were retained for SNP calling. The final matrix consisted of 38,273 high quality SNPs, which were used for subsequent phylogenetic and divergence time analyses.

**Phylogenetic Relationships of *S. sieboldii*:** Phylogenetic analyses were conducted based on the nuclear SNP dataset. First, the best - fit nucleotide substitution model was selected using JMODELTEST v2.1.1 (Posada, 2008) based on the Akaike Information Criterion (AIC). Phylogenetic reconstruction was then performed using the Maximum Likelihood (ML) method implemented in RAxML-NG (Kozlov et al., 2019), with 1,000 bootstrap replicates. The resulting phylogenetic tree was visualized and exported using FIGTREE v1.4.3 (<http://tree.bio.ed.ac.uk/software/figtree/>). The ML analyses revealed a phylogenetic pattern consistent with geographical distributions (**Fig. S1** in Appendix). Two distinct clades, corresponding to western and eastern regions, were strongly supported (bootstrap support [BS] = 100), indicating an early divergence across the East China Sea and the Yellow - Bohai Sea. The western clade included nearly all populations from China (except one population in Liaoning east of the seaway) and further split into two subclades: one representing the "Red" gene pool in south eastern China (including Taiwan Island), and the other corresponding to admixed gene pools in north eastern China. The eastern clade was further divided by the Korea - Tsushima Strait and the Itoigawa - Shizuoka Tectonic Line, two well - known biogeographical barriers. Populations with the "yellow" gene pool in north Japan formed one subclade, while those with admixed gene pools in south Japan showed closer relationships with populations from Korea and nearby Liaoning of north eastern China, which belong to the "Blue" gene pool (**Fig. S1** in Appendix, detailed descriptions of the gene pools are provided in **Fig. 1**).

**Divergence Time Estimation of Lineages of *S. sieboldii*:** Divergence times were estimated using BEAST 1.8.3 (Drummond et al., 2012) with an uncorrelated lognormal relaxed clock model and estimated mutation rates. The divergence time between *S. sieboldii* and its sister species, *S. scobinicaulis*, was derived from a previous fossil - constrained analyses of the Smilacaceae family (Qi et al., 2023). A calibration constraint of 5.06 Ma (95% HPD: 2.51 - 6.89 Ma) with a lognormal distribution was applied. Four independent Markov chain Monte Carlo (MCMC) runs were conducted, each with 50,000,000 generations and parameters sampled every 5,000 generations. Trees from the different runs were combined using LOGCOBINER 1.8.3 (Drummond et al., 2012), after discarding the first 20% of generations as burn-in. A maximum clade credibility tree was generated with a posterior probability limit of 0.5 using TREEANNOTATOR 1.8.3 (Drummond et al., 2012) and visualized in FIGTREE v1.4.3. The BEAST - derived chronogram of Smilacaceae, based on fossil calibration points, suggested that *S. sieboldii* and *S. scobinicaulis* diverged in the early Pliocene (Node A, 5.24 Ma, 95% HPD: 3.24 - 7.39 Ma). Intraspecific divergence intensified around the Mio - Pliocene boundary. The first divergence occurred across the East China Sea, separating the China clade (West) from the Liaoning - Korea - Japan clade (East) at 2.79 Ma (Node B, 95% HPD: 1.63 - 3.97 Ma). Within the China clade, the China Mainland and Taiwan lineages likely diverged across the Taiwan Strait during the early Pleistocene (Node D, 2.49 Ma, 95% HPD: 1.46 - 3.57 Ma). Simultaneously, the Liaoning & Korean and Japanese lineages diverged across the Korea - Tsushima Strait at 2.38 Ma (Node C, 95% HPD: 1.35 - 3.37 Ma). More recent diversification events occurred between eastern and northern China (2.15 Ma), as well as between southern and northern Japan (1.97 Ma).


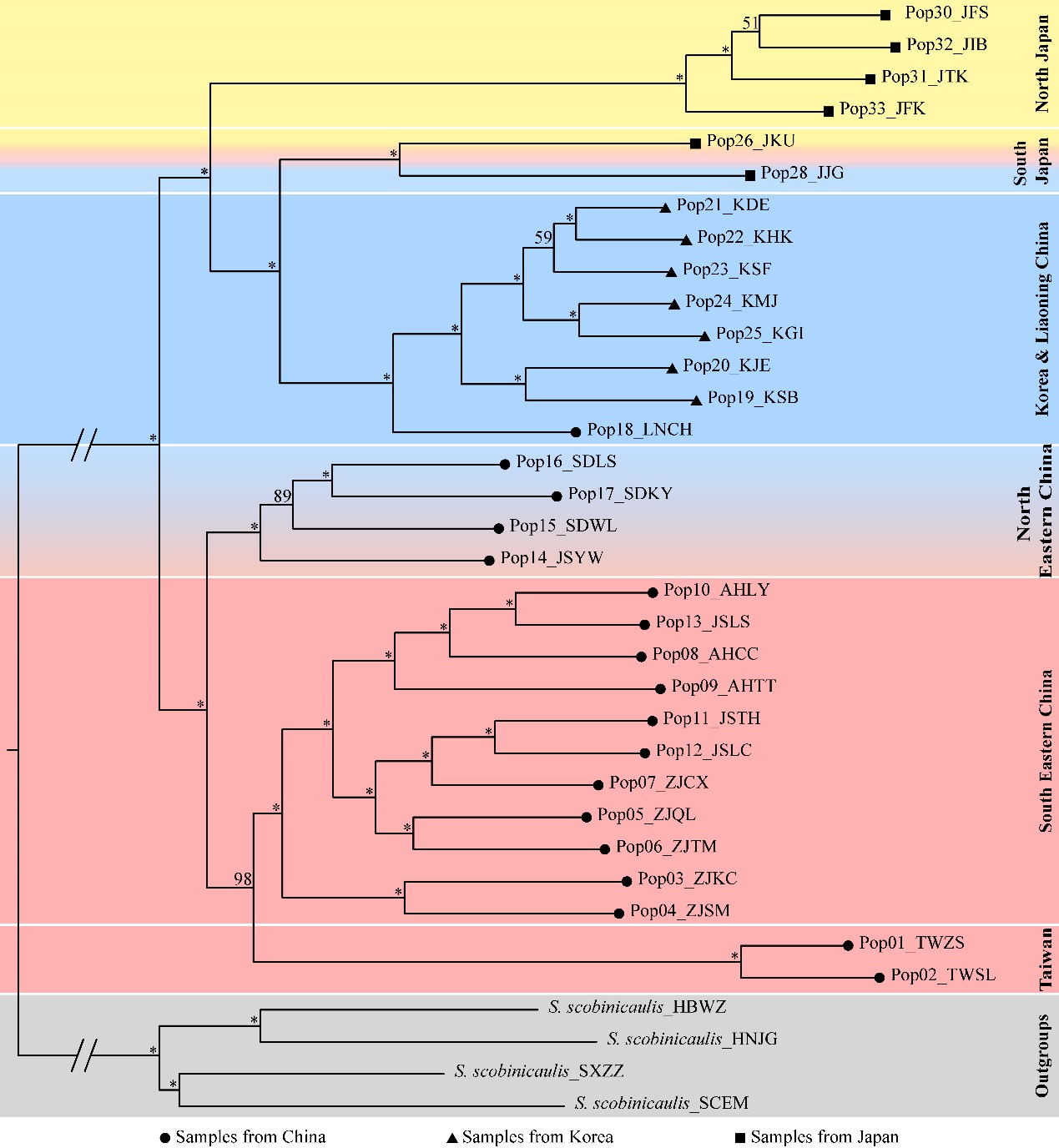


**Fig. S1** Phylogenetic tree resulting from Maximum Likelihood analyses based on genome-wide SNPs. Bootstrap values = 100 are indicated as “*” on the corresponding branches. *Smilax scobinicaulis* was set as outgroup to root the tree.


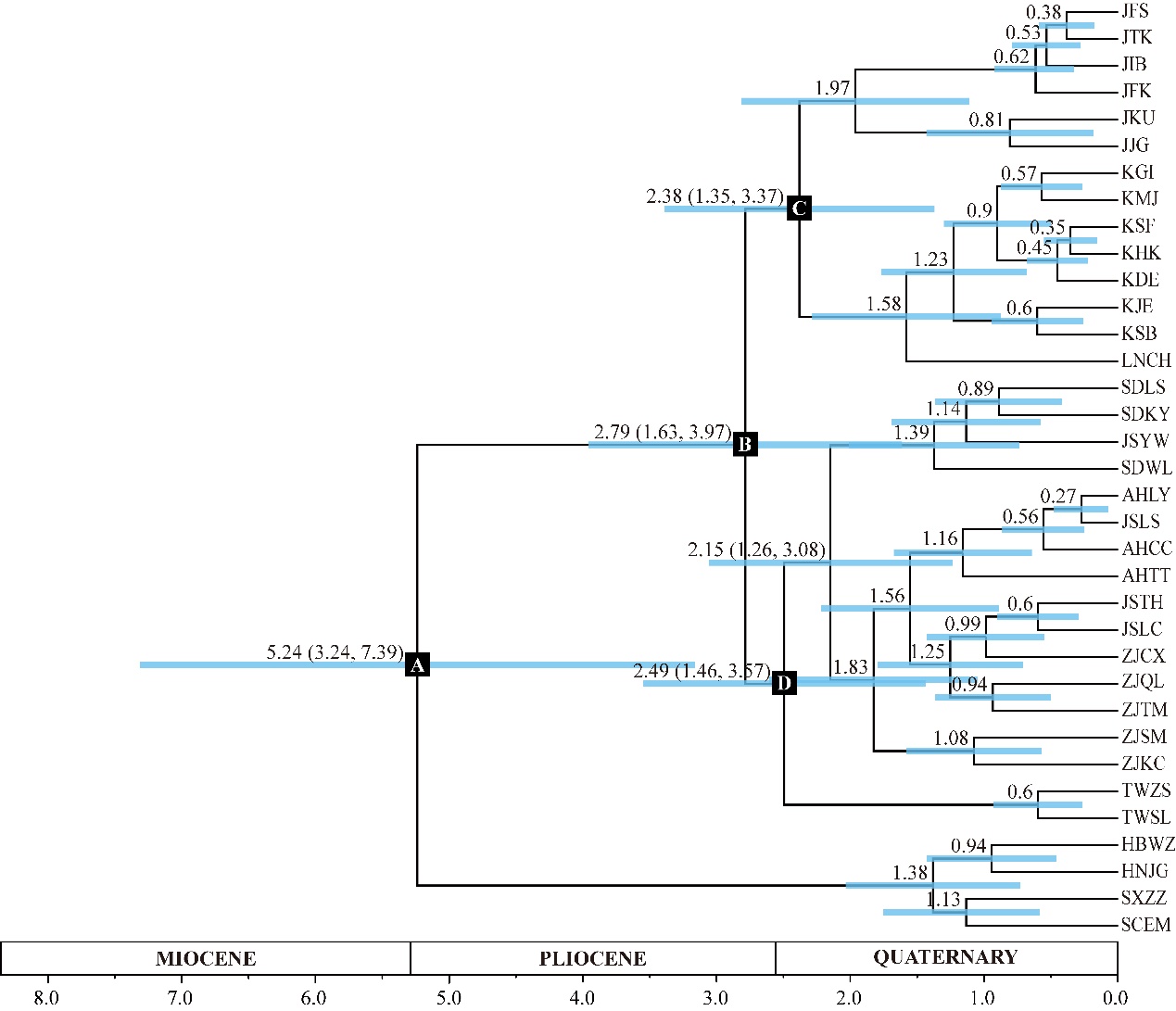


**Fig. S2** Divergence times of the *Smilax sieboldii* lineages estimated based on the nuclear SNP matrix: Node A, the secondary calibration point derived from Qi et al., 2023; Node B, divergence time between the China clade and Lianing – Korea - Japan clade; Node C, divergence time between the Korean and Japanese lineages; Node D, divergence time between the mainland China and Taiwan lineages. The divergence times for other nodes are indicated by numerical values, with blue bars representing the 95% confidence intervals.


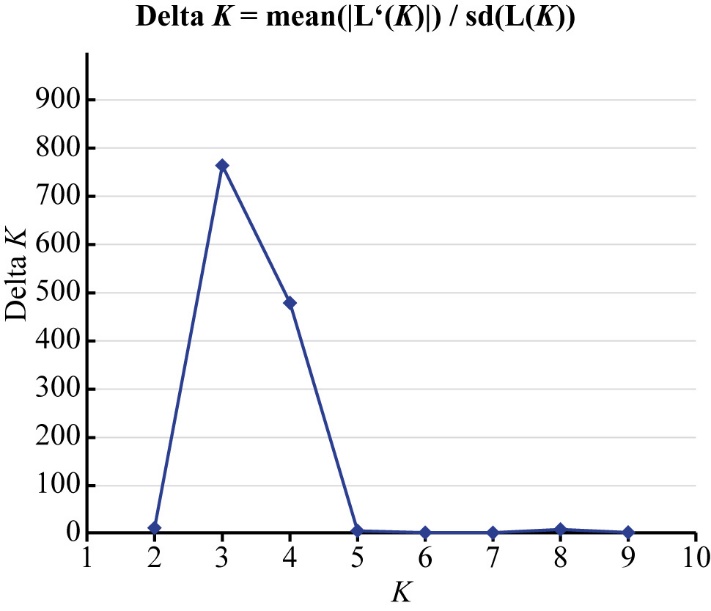


**Fig. S3** Delta K values identified by Evanno statistics (Evanno et al., 2005) in STRUCTURE HARVESTER.


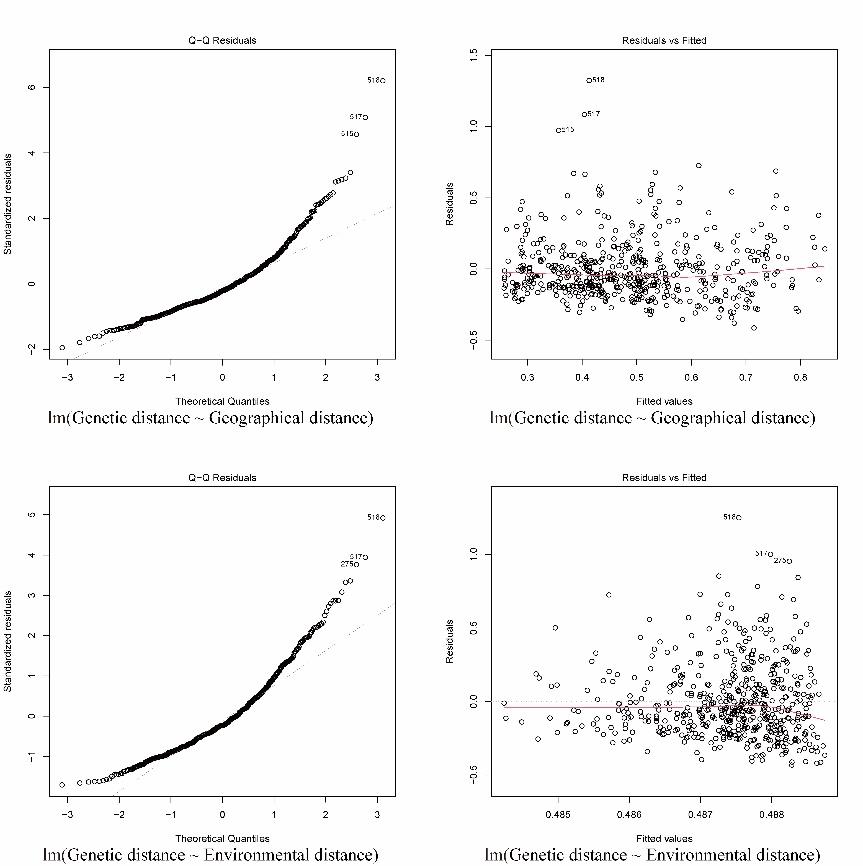


**Fig. S4** Regression diagnostics: Q-Q plots and residuals vs. fitted values

**Table S1** Information of plant materials used in this study.

| **Sample ID** | **Collector** | **Voucher Num.** | **Collect Date** | **Collect Locality** | **Lon. & Lat.** | **Altitude** | **Num. of Samples for SSR** | **Num. of Samples for RAD-seq** |
| --- | --- | --- | --- | --- | --- | --- | --- | --- |
| **Species: *Smilax sieboldii* Miq.** | |  |  |  |  |  |  |  |
| Pop02_TWSL | Yunpeng Zhao | 14324 | 2015.11.25 | Shanlinxi, Nantou, Taiwan, China | E120.795, N23.631 | 1696 | **15** | 1 |
| Pop04_ZJSM | Minqi Cai | 330281150429947 | 2014.04.29 | Siming Mountain, Yuyao, Zhejiang, China | E121.084, N29.745 | 909 | 10 | 1 |
| Pop06_ZJTM | Wenwu Zhang | 081003001-8 | 2008.10.03 | Tianmu Mountain, Linan, Zhejiang, China | E119.424, N30.349 | 1480 | 8 | 1 |
| Pop07_ZJCX | Yunpeng Zhao et al. | HZU00529-534 | 2015.10.06 | Xiaopu, Changxing, Zhejiang, China | E119.711, N31.023 | 289 | 12 | 1 |
| Pop08_AHCC | Yunpeng Zhao et al. | 14206 | 2015.05.17 | Changchong, Tongling, Anhui,China | E118.042, N30.900 | 248 | 9 | 1 |
| Pop10_AHLY | Yalu Ru | Ru150928003 | 2015.09.28 | Langya Mountain, Chuzhou, Anhui,China | E118.280, N32.281 | 110 | 10 | 1 |
| Pop11_JSTH | Yunpeng Zhao | 91004 | 2009.10.19 | Taihua Mountain, Yixing, Jiangsu, China | E119.617, N31.167 | 110 | 11 | 1 |
| Pop12_JSLC | Yunpeng Zhao et al. | HZU00441-9 | 2015.10.04 | Longchi Mountain, Yixing, Jiangsu, China | E119.746, N31.248 | 226 | 12 | 1 |
| Pop18_LNCH | Pan Li | LP0809LN | 2008.09.01 | Changhai, Liaoning, China | E122.340, N39.160 | 50 | 12 | 1 |
| Pop19_KSB | Joongku Lee | Korea03 | 2009.06.24 | Andeok-myeon, Seogwipo-si, Jeju-do, Korea | E126.306, N33.240 | 84-118 | 10 | 1 |
| Pop20_KJE | Joongku Lee | Korea01 | 2009.06.24 | Hallasan, Seogwipos, Jeju-do, Korea | E126.458, N33.355 | 1082 | 9 | 1 |
| Pop23_KSF | Chengxin Fu | Fu151021001 | 2015.10.21 | Korean Folk Village, Yongin-si, Gyeonggi-do, Korea | E127.119, N37.269 | 130 | 7 | 1 |
| Pop24_KMJ | Joongku Lee | GG13-GG27 | 2009.10.08 | Myeongjisan, Gapyeong-gun, Gyeonggi-do, Korea | E127.473, N37.935 | 213-369 | 14 | 1 |
| Pop25_KGI | Joongku Lee | GG01-GG12 | 2009.10.07 | Gwangdeoksan, Pocheon-si, Gyeonggi-do, Korea | E127.442, N38.105 | 620-766 | 12 | 1 |
| Pop26_JKU | Yihui Liu | Kum | 2009.06.24 | Miyazaki ken, Kyushu, Japan | E131.178, N32.716 | 550 | 8 | 1 |
| Pop28_JJG | Yihui Liu | Jgn | 2009.06.19 | Jigangjisan, Tokushima ken, Japan | E134.332, N34.138 | 541 | 7 | 1 |
| Pop29_JNR | Akiyo Naiki | JP091012 | 2009.10.12 | Nara ken, Japan | E135.867, N34.267 | 910 | 8 | 1 |
| Pop30_JFS | Chengxin Fu et al | 201505092 | 2015.05.09 | Fuji Mountain,Shizuoka ken, Japan | E138.770, N35.503 | 877 | 9 | 1 |
| Pop31_JTK | Pan Li | BQ0907232 | 2009.07.04 | Okutama machi, Tokyo, Japan | E139.109, N35.809 | 638 | 14 | 1 |
| Pop32_JIB | Xiaoling Yan | JP0905001 | 2009.05.21 | Kashima Jingu, Ibaraki ken, Japan | E140.619, N36.553 | 51 | 16 | 1 |
| Pop33_JFK | Xiaoling Yan | JP0905002 | 2009.05.24 | Sugitu, Fukushima ken, Japan | E140.402, N37.557 | 220 | 14 | 1 |
| Pop03_ZJKC | Ming Jiang | JM091001001 | 2009.11.01 | Kuocang Mountain, Taizhou, Zhejiang, China | E120.914, N28.797 | 1195 | 14 | 1 |
| Pop22_KHK | Joongku Lee | GB01-15 | 2009.09.04 | Seohu-myeon, Andong-si, Gyeongsangbuk-do, Korea | E128.619, N36.656 | 632-653 | 12 | 1 |
| Pop01_TWZS | Xiaoxian Li | 812003 | 2008.12.00 | Zhu Mountain, Jiayi, Taiwan, China | E120.500, N23.310 | 2218 | 6 | 1 |
| Pop05_ZJQL | Yalu Ru | Ru150921001 | 2015.09.21 | Qingliang Mountain, Linan, Zhejiang, China | E119.123, N30.302 | 1450 | 11 | 1 |
| Pop09_AHTT | Pan Li | BQ0906222 | 2009.06.21 | Tiantang, Jinzhai, Anhui,China | E115.450, N31.100 | 945 | 14 | 1 |
| Pop27_JOM | Yihui Liu | omo | 2009.06.21 | Ehime ken, Japan | E132.771, N33.668 | 200-500 | 13 | 1 |
| Pop13_JSLS | Yunpeng Zhao et al. | 141001-141017 | 2014.10.11 | Lao Mountain, Nianjin, Jiangsu, China | E118.546, N32.075 | 125 | 11 | 1 |
| Pop14_JSYW | Yalu Ru | Ru20150512001 | 2015.05.12 | Yuwan, Lianyungang, Jiangsu, China | E119.334, N34.642 | 54-114 | 11 | 1 |
| Pop16_SDLS | Yao Chen | CY160057 | 2016.05.18 | Lao Mountain, QingDao, Shandong, China | E120.502, N36.238 | 214 | 8 | 1 |
| Pop17_SDKY | Ying Wan | KY0906002w | 2009.07.07 | Kunyu Mountain, Yantai, Shandong, China | E121.767, N37.233 | 689 | 12 | 1 |
| Pop21_KDE | Joongku Lee | JB01~15 | 2009.05.30 | Deogyusan, Muju-gun, Jeollabuk-do, Korea | E127.683, N35.833 | 524-550 | 12 | 1 |
| Pop15_SDWL | Yalu Ru | Ru20150515001 | 2015.05.15 | Wulian Mountain, Rizhao, Shandong, China | E119.386, N35.694 | 124-409 | 12 | 1 |
| **Species: *S. scobinicaulis* C.H.Wright** | |  |  |  |  |  |  |  |
| SCEM | Pan Li | LP150885 | 2015.10.12 | Emei Mountain, Sichuan, China | E103.360, N29.550 | 1674 | 0 | 1 |
| HBWZ | Pan Li | LP150444 | 2015.08.06 | Wuzhi Mountain, Hubei, China | E110.884, N31.090 | 1591 | 0 | 1 |
| SXZZ | Pan Li | LP150524 | 2015.08.16 | Zhouzhi, Shanxi, China | E108.153, N33.868 | 1422 | 0 | 1 |
| HNJG | Yalu Ru | Ru016 | 2015.10.16 | Jigong Mountain, Henan, China | E114.072, N31.812 | 492-552 | 0 | 1 |

**Table S1** – (continued)

| **Sample ID** | **Number of alleles** | **Number of private alleles** | **Allelic richness** | **Observed heterozygosity** | **Expected heterozygosity** | **Wright’s inbreeding coefficient (*Fis*)** |
| --- | --- | --- | --- | --- | --- | --- |
| **Species: *Smilax sieboldii* Miq.** | |  |  |  |  |  |
| Pop02_TWSL | 48 | 0 | 2.89 | 0.51 | 0.48 | -0.0663 |
| Pop04_ZJSM | 48 | 0 | 3.15 | 0.65 | 0.53 | -0.2183 |
| Pop06_ZJTM | 56 | 0 | 3.71 | 0.81 | 0.66 | -0.2314 |
| Pop07_ZJCX | 58 | 0 | 3.48 | 0.65 | 0.6 | -0.0872 |
| Pop08_AHCC | 42 | 0 | 2.92 | 0.73 | 0.56 | -0.3185 |
| Pop10_AHLY | 36 | 0 | 2.46 | 0.59 | 0.43 | -0.361 |
| Pop11_JSTH | 51 | 0 | 3.29 | 0.63 | 0.57 | -0.0917 |
| Pop12_JSLC | 52 | 0 | 3.38 | 0.73 | 0.58 | -0.2401 |
| Pop18_LNCH | 36 | 0 | 2.54 | 0.6 | 0.49 | -0.2227 |
| Pop19_KSB | 44 | 0 | 2.89 | 0.51 | 0.5 | -0.0252 |
| Pop20_KJE | 48 | 0 | 3.17 | 0.68 | 0.58 | -0.1742 |
| Pop23_KSF | 42 | 0 | 2.89 | 0.55 | 0.47 | -0.1618 |
| Pop24_KMJ | 51 | 0 | 3.21 | 0.69 | 0.57 | -0.2164 |
| Pop25_KGI | 44 | 0 | 2.92 | 0.56 | 0.51 | -0.09 |
| Pop26_JKU | 41 | 0 | 2.84 | 0.68 | 0.53 | -0.2821 |
| Pop28_JJG | 25 | 0 | 1.82 | 0.3 | 0.24 | -0.25 |
| Pop29_JNR | 31 | 0 | 2.35 | 0.52 | 0.44 | -0.1765 |
| Pop30_JFS | 34 | 0 | 2.35 | 0.47 | 0.38 | -0.2277 |
| Pop31_JTK | 52 | 0 | 3 | 0.5 | 0.48 | -0.0304 |
| Pop32_JIB | 29 | 0 | 2.14 | 0.4 | 0.32 | -0.2616 |
| Pop33_JFK | 29 | 0 | 1.96 | 0.37 | 0.27 | -0.3718 |
| Pop03_ZJKC | 58 | 1 | 3.28 | 0.65 | 0.56 | -0.1751 |
| Pop22_KHK | 51 | 1 | 3.11 | 0.55 | 0.54 | -0.0218 |
| Pop01_TWZS | 47 | 2 | 3.39 | 0.69 | 0.62 | -0.1236 |
| Pop05_ZJQL | 70 | 2 | 4.27 | 0.73 | 0.69 | -0.058 |
| Pop09_AHTT | 61 | 2 | 3.73 | 0.71 | 0.63 | -0.1156 |
| Pop27_JOM | 54 | 2 | 3.26 | 0.55 | 0.51 | -0.0637 |
| Pop13_JSLS | 44 | 3 | 2.93 | 0.65 | 0.56 | -0.1547 |
| Pop14_JSYW | 56 | 3 | 3.48 | 0.62 | 0.6 | -0.0331 |
| Pop16_SDLS | 43 | 3 | 3 | 0.73 | 0.55 | -0.3244 |
| Pop17_SDKY | 51 | 3 | 3.27 | 0.66 | 0.58 | -0.1488 |
| Pop21_KDE | 47 | 4 | 2.82 | 0.44 | 0.44 | -0.002 |
| Pop15_SDWL | 70 | 5 | 4.11 | 0.75 | 0.68 | -0.0967 |
| **Species: *S. scobinicaulis* C.H.Wright** | |  |  |  |  |  |
| SCEM | - | - | - | - | - | - |
| HBWZ | - | - | - | - | - | - |
| SXZZ | - | - | - | - | - | - |
| HNJG | - | - | - | - | - | - |

**Table S1** – (continued)

| **Sample ID** | bio1 | bio2 | bio3 | bio4 | bio5 | bio6 | bio7 | bio8 | bio9 | bio10 | bio11 | bio12 | bio13 | bio14 | bio15 | bio16 | bio17 | bio18 | bio19 |
| --- | --- | --- | --- | --- | --- | --- | --- | --- | --- | --- | --- | --- | --- | --- | --- | --- | --- | --- | --- |
| **Species: *Smilax sieboldii* Miq.** | |  |  |  |  |  |  |  |  |  |  |  |  |  |  |  |  |  |  |
| Pop02_TWSL | 21.06667 | 6.033333 | 36.12774 | 383.2773 | 28.5 | 11.8 | 16.7 | 25.15 | 17.08333 | 25.15 | 15.85 | 2722 | 594 | 19 | 97.84802 | 1618 | 65 | 1618 | 99 |
| Pop04_ZJSM | 14.27083 | 5.641667 | 37.36203 | 335.6978 | 20.9 | 5.8 | 15.1 | 17.85 | 10.75 | 17.85 | 9.633333 | 2294 | 451 | 35 | 77.34177 | 1175 | 109 | 1175 | 150 |
| Pop06_ZJTM | 11.71667 | 6.733334 | 23.87707 | 764.5805 | 25.5 | -2.7 | 28.2 | 20.86667 | 2.083333 | 20.86667 | 2.083333 | 1926 | 301 | 52 | 52.73413 | 763 | 201 | 763 | 201 |
| Pop07_ZJCX | 12.35 | 7.1 | 23.90572 | 809.0061 | 26.9 | -2.8 | 29.7 | 22.05 | 4.333333 | 22.05 | 2.15 | 1542 | 222 | 45 | 48.39616 | 588 | 168 | 588 | 177 |
| Pop08_AHCC | 11.18333 | 7.3 | 24.74576 | 808.5659 | 25.4 | -4.1 | 29.5 | 18.86667 | 2.916667 | 20.9 | 0.933333 | 1676 | 262 | 43 | 49.8694 | 668 | 167 | 660 | 183 |
| Pop10_AHLY | 10.47917 | 7.158333 | 24.51484 | 803.174 | 24.6 | -4.6 | 29.2 | 20.13333 | 2.366667 | 20.13333 | 0.333333 | 1636 | 250 | 43 | 49.53241 | 647 | 166 | 647 | 179 |
| Pop11_JSTH | 14.49167 | 7.4 | 23.87097 | 871.0178 | 29.8 | -1.2 | 31 | 24.98333 | 5.716667 | 24.98333 | 3.55 | 1208 | 200 | 31 | 54.95461 | 516 | 121 | 516 | 137 |
| Pop12_JSLC | 15.57917 | 7.791667 | 24.8935 | 878.1941 | 31 | -0.3 | 31.3 | 23.85 | 6.45 | 26.11667 | 4.5 | 1360 | 236 | 30 | 52.29937 | 560 | 132 | 541 | 136 |
| Pop18_LNCH | 15.125 | 8.766666 | 27.05761 | 884.7098 | 30.7 | -1.7 | 32.4 | 23.68333 | 5.766667 | 25.73333 | 3.883333 | 1371 | 229 | 28 | 59.15292 | 615 | 120 | 608 | 120 |
| Pop19_KSB | 14.95417 | 8.375 | 25.76923 | 912.9858 | 30.5 | -2 | 32.5 | 25.88333 | 3.366667 | 25.88333 | 3.366667 | 986 | 204 | 27 | 62.40476 | 464 | 98 | 464 | 98 |
| Pop20_KJE | 14.9 | 7.35 | 23.55769 | 880.6531 | 30.3 | -0.9 | 31.2 | 25.5 | 6 | 25.5 | 3.816667 | 1164 | 189 | 30 | 53.39023 | 493 | 120 | 493 | 132 |
| Pop23_KSF | 14.575 | 7.8 | 24.60568 | 882.3174 | 30.1 | -1.6 | 31.7 | 25.23333 | 5.716667 | 25.23333 | 3.516667 | 1155 | 186 | 32 | 53.57662 | 495 | 123 | 495 | 136 |
| Pop24_KMJ | 14.76667 | 8.183333 | 25.2572 | 911.6277 | 30.4 | -2 | 32.4 | 25.7 | 3.233333 | 25.7 | 3.233333 | 1042 | 202 | 26 | 59.31406 | 477 | 103 | 477 | 103 |
| Pop25_KGI | 12.85 | 8.433333 | 25.55556 | 936.2862 | 28.3 | -4.7 | 33 | 23.96667 | 0.95 | 23.96667 | 0.95 | 872 | 231 | 12 | 96.17274 | 523 | 46 | 523 | 46 |
| Pop26_JKU | 12.125 | 8.816667 | 25.77973 | 969.8793 | 27.9 | -6.3 | 34.2 | 23.45 | -0.36667 | 23.45 | -0.36667 | 818 | 228 | 9 | 101.2045 | 502 | 32 | 502 | 32 |
| Pop28_JJG | 12.18333 | 6.95 | 21.65109 | 940.3416 | 27.4 | -4.7 | 32.1 | 23.06667 | 0.166667 | 23.18333 | 0.166667 | 699 | 165 | 10 | 90.72952 | 403 | 33 | 399 | 33 |
| Pop29_JNR | 10.22917 | 6.158333 | 19.67519 | 929.5073 | 25.3 | -6 | 31.3 | 21.16667 | -1.45 | 21.16667 | -1.45 | 747 | 190 | 11 | 95.44467 | 449 | 42 | 449 | 42 |
| Pop30_JFS | 10.07083 | 7.491667 | 21.10329 | 1040.888 | 26.4 | -9.1 | 35.5 | 22 | -3.35 | 22 | -3.35 | 679 | 182 | 8 | 101.8724 | 421 | 26 | 421 | 26 |
| Pop31_JTK | 15.75833 | 6.599999 | 24.81203 | 733.9459 | 29.4 | 2.8 | 26.6 | 24.36667 | 9.083333 | 24.98333 | 6.9 | 1751 | 280 | 49 | 56.07779 | 779 | 188 | 690 | 190 |
| Pop32_JIB | 9.8875 | 6.491667 | 23.60606 | 776.1242 | 23.7 | -3.8 | 27.5 | 19.01667 | 2.6 | 19.38333 | 0.3 | 2008 | 338 | 57 | 57.70601 | 918 | 214 | 818 | 220 |
| Pop33_JFK | 9.383333 | 10.15 | 28.27298 | 973.4202 | 26.3 | -9.6 | 35.9 | 20.96667 | -3.1 | 20.96667 | -3.1 | 1392 | 322 | 29 | 82.62716 | 774 | 102 | 774 | 102 |
| Pop03_ZJKC | 10.49583 | 11.04167 | 29.92322 | 976.3323 | 27.9 | -9 | 36.9 | 21.55 | -1.96667 | 22.16667 | -1.96667 | 1205 | 268 | 26 | 80.14246 | 648 | 91 | 645 | 91 |
| Pop22_KHK | 11.3 | 9.816667 | 26.24777 | 1033.472 | 28.8 | -8.6 | 37.4 | 23.08333 | -1.93333 | 23.58333 | -1.93333 | 1319 | 337 | 24 | 93.01196 | 772 | 77 | 758 | 77 |
| Pop01_TWZS | 8.695833 | 9.908334 | 26.07456 | 1041.776 | 26.1 | -11.9 | 38 | 20.38333 | -4.78333 | 20.96667 | -4.78333 | 1298 | 353 | 16 | 102.0914 | 802 | 60 | 789 | 60 |
| Pop05_ZJQL | 7.525 | 10.18333 | 26.58834 | 1041.017 | 25 | -13.3 | 38.3 | 19.21667 | -5.95 | 19.8 | -5.95 | 1319 | 359 | 18 | 100.4995 | 806 | 67 | 796 | 67 |
| Pop09_AHTT | 12.94583 | 8.041666 | 27.25989 | 796.8759 | 27.4 | -2.1 | 29.5 | 22.23333 | 5.316667 | 22.61667 | 3.016667 | 2426 | 436 | 62 | 63.31868 | 1144 | 215 | 963 | 233 |
| Pop27_JOM | 13.01667 | 7.45 | 25.42662 | 805.8376 | 27.6 | -1.7 | 29.3 | 19.65 | 5.466667 | 22.9 | 3.1 | 2051 | 321 | 62 | 52.14399 | 843 | 214 | 736 | 214 |
| Pop13_JSLS | 13.74167 | 7.7 | 25.92593 | 814.7806 | 28.8 | -0.9 | 29.7 | 20.51667 | 3.816667 | 23.76667 | 3.816667 | 1775 | 275 | 41 | 54.71292 | 675 | 154 | 669 | 154 |
| Pop14_JSYW | 10.2125 | 7.908333 | 26.62738 | 809.947 | 25.2 | -4.5 | 29.7 | 19.76667 | 0.35 | 20.13333 | 0.35 | 2195 | 315 | 62 | 48.60813 | 849 | 221 | 823 | 221 |
| Pop16_SDLS | 11.36667 | 10.51667 | 32.25972 | 788.8436 | 27.3 | -5.3 | 32.6 | 21.01667 | 1.783333 | 21.01667 | 1.783333 | 1519 | 242 | 39 | 54.04817 | 632 | 140 | 632 | 140 |
| Pop17_SDKY | 12.025 | 9.4 | 29.28349 | 807.033 | 27.9 | -4.2 | 32.1 | 21.93333 | 2.266667 | 21.93333 | 2.266667 | 1364 | 209 | 30 | 56.88066 | 571 | 112 | 571 | 112 |
| Pop21_KDE | 13.1375 | 8.408333 | 28.59977 | 753.5254 | 27.6 | -1.8 | 29.4 | 18.85 | 4.083333 | 22.55 | 4.083333 | 1463 | 185 | 36 | 44.8907 | 506 | 147 | 499 | 147 |
| Pop15_SDWL | 11.73333 | 8.916666 | 28.2173 | 845.7496 | 28.3 | -3.3 | 31.6 | 22.08333 | 1.633333 | 22.08333 | 1.633333 | 1150 | 160 | 46 | 43.4044 | 452 | 147 | 452 | 147 |
| **Species: *S. scobinicaulis* C.H.Wright** | | |  |  |  |  |  |  |  |  |  |  |  |  |  |  |  |  |  |
| SCEM | - | - | - | - | - | - |  |  |  |  |  |  |  |  |  |  |  |  |  |
| HBWZ | - | - | - | - | - | - |  |  |  |  |  |  |  |  |  |  |  |  |  |
| SXZZ | - | - | - | - | - | - |  |  |  |  |  |  |  |  |  |  |  |  |  |
| HNJG | - | - | - | - | - | - |  |  |  |  |  |  |  |  |  |  |  |  |  |

* bio1 = Annual Mean Temperature; bio2 = Mean Diurnal Range (Mean of monthly (max temp - min temp)); bio3 = Isothermality (bio2/bio7) (×100); bio4 = Temperature Seasonality (standard deviation ×100); bio5 = Max Temperature of Warmest Month; bio6 = Min Temperature of Coldest Month; bio7 = Temperature Annual Range (bio5-bio6); bio8 = Mean Temperature of Wettest Quarter; bio9 = Mean Temperature of Driest Quarter; bio10 = Mean Temperature of Warmest Quarter; bio11 = Mean Temperature of Coldest Quarter; bio12 = Annual Precipitation; bio13 = Precipitation of Wettest Month; bio14 = Precipitation of Driest Month; bio15 = Precipitation Seasonality (Coefficient of Variation); bio16 = Precipitation of Wettest Quarter; bio17 = Precipitation of Driest Quarter; bio18 = Precipitation of Warmest Quarter; bio19 = Precipitation of Coldest Quarter.
